# The New York State Medical Association

**Published:** 1886-12

**Authors:** 


The New York State Medical Association.—This Society held its third annual meeting in New York last month as announced. The number in attendance was considerably less than last year, but the work accomplished was fully as satisfactory and of a highly scientific order. The papers announced were nearly all presented and were in good order to go to the publishing committee. This committee, of which Dr. Cronyn is chairman, hope to be able to issue the complete volume of the transactions in three months. It will contain many valuable contributions. Dr. Isaac Taylor, of New York, was elected president.

---

The *Scientific American*, published by Munn & Co., New York, during forty years, is, beyond all question, the leading paper relating to science, mechanics and inventions, published on this continent. Each weekly issue presents the latest scientific topics in an interesting and reliable manner, accompanied with engravings prepared expressly to demonstrate the subjects. The *Scientific American* is invaluable to every person desiring to keep pace with the inventions and discoveries of the day.

